# Effect of flavonoids on skeletal muscle mass, strength and physical performance in middle-aged and older adults with or without Sarcopenia: A meta-analysis of randomized controlled trials

**DOI:** 10.3389/fnut.2022.1013449

**Published:** 2022-10-10

**Authors:** Yuzhuo Li, Yun Liu, Rongshao Tan, Yan Liu

**Affiliations:** ^1^Department of Nephrology, Guangzhou Red Cross Hospital, Jinan University, Guangzhou, China; ^2^Guangzhou Institute of Disease-Oriented Nutritional Research, Guangzhou Red Cross Hospital, Jinan University, Guangzhou, China

**Keywords:** middle-age, elderly, flavonoids, muscle mass, muscle strength, physical performance, Sarcopenia

## Abstract

The role of flavonoids in regulating the synthesis and function of skeletal muscles is increasingly recognized. However, randomized controlled trials have yielded inconsistent results on the influence of flavonoids on human muscular parameters. Therefore, we performed a meta-analysis to evaluate the possible effects of flavonoids on sarcopenia-related parameters in middle-aged and elderly people. Eligible literature and randomized controlled trials reports have been extensively searched from PubMed, Cochrane Library, Web of Science, and EMBASE databases until April 2022. A total of 20 articles involving 796 participants were available for the meta-analysis. There were significant benefits for participants in appendicular muscle mass gain (SMD = 0.29; 95% CI: 0.07, 0.52; *P* = 0.01) and 6-min walk distance (SMD = 0.37; 95% CI: 0.01, 0.73; *P* = 0.05). A subgroup analysis indicated that flavonoid significantly improves appendicular muscle mass (SMD = 0.50; 95% CI: 0.21, 0.80; *P* < 0.01) and Timed-Up and Go test (SMD = −0.47; 95% CI: −0.85, −0.09; *P* = 0.02) in Sarcopenia population. Our results provide insight into the effects of flavonoids on skeletal muscle mass and gait speed for those without exercise. However, there was no significant improvement in the subjects' muscle strength.

**Systematic review registration:**

https://www.crd.york.ac.uk/prospero/display_record.php?RecordID=334383, identifier: CRD42022334383.

## Introduction

Skeletal muscle is the most extensive mass system of the organ in the body, which plays a critical role in maintaining physical functioning and metabolic health (1). With aging, there is an unavoidable loss of muscle mass and strength (2). Muscle mass loss occurs at approximately 1–2% per year from middle age, and muscle strength decreases by 1.5% per year from 40 years of age and 3% per year thereafter (3). Such a trend is associated with increased adverse outcomes, including falls, functional decline, frailty, and mortality (4, 5), which translates into higher healthcare costs (6). In clinical practice, the EWGSOP2 (European Working Group on Sarcopenia in Older People) states that a person with low muscle mass and low muscle strength or quality will be diagnosed with Sarcopenia (7). Sarcopenia has been already recognized as an independent condition by an ICD-10-CM code in 2016 (8).

Flavonoids are polyphenolic phytochemicals distributed commonly in different fruits, vegetables, plants, and herbs, including anthocyanidins (such as cyandin, delphinidin, and malvidin, found in colored berries and red wine), flavan-3-ols (such as catechin, epicatechin, theaflavin, etc., found in cocoa, apples, and grapes), flavanones (such as eriodictyol, hesperidin, and naringenin exclusive of citrus fruits), flavones (such as apigenin and luteolin, commonly found in celery, parsley, and chamomile tea), flavonols (such as kaempferol, quercetin, and myricetin, found in tea, broccoli, and various fruits), and isoflavones (such as daidzein, genistein and glycitein, present mainly in soy and soy products) (9). Flavonoids are widely known for their anti-inflammatory and antioxidant properties. Current evidence indicates that several sub-classes of flavonoids and their primary food sources may regulate metabolism in skeletal muscle that acts as the prior site of glucose storage (10). Flavonoids could also preserve muscle structure and function directly through physiological mechanisms or indirectly through distinct molecular signal pathways (11, 12).

Although both *in vitro* and *in vivo* studies have shown the association of flavonoids with skeletal muscle mass, clinical findings have been inconsistent concerning the relationship between flavonoids and Sarcopenia (13, 14). Therefore, this systematic review aims to evaluate the current evidence of flavonoid intake/supplementation on skeletal muscle mass, strength, and physical performance in middle-aged and older adults with or without Sarcopenia, and draw research-based conclusions on the practical implications of the findings.

## Materials and methods

This systematic review followed the guidelines recommended by the Preferred Reporting Items for Systematic Reviews and Meta-Analysis (PRISMA) (15). The protocol for this study was registered at PROSPERO (Registration Number: CRD42022334383).

### Searches strategy

We searched PubMed, Embase, Web of Science, and the Cochrane Library (Cochrane Central Register of Controlled Trials) in English, from the date of inception until 15 April 2022.

The following Medical Subject Heading (MeSH) terms and keywords were used to search the studies: “middle-aged,” “elderly,” “skeletal muscle,” “muscle performance,” “muscle strength,” “dynapenia,” “frailty,” “sarcopenia,” “flavonoids,” “flavonols,” “flavones,” “flavanones,” “isoflavones,” and “anthocyanidins” (Supplementary Table 1).

Two reviewers (YZL and YL) independently reviewed all relevant articles. Disagreements were resolved by group discussion.

### Selection criteria

Following the removal of duplicate articles, two reviewers (YZL and YL) independently screened titles and abstracts for eligibility using the inclusion and exclusion criteria discussed below. Disagreements were discussed by the reviewers and resolved through consensus.

Trials included in the meta-analysis must meet the following criteria: (1) The participants included adults in middle-age and elderly, with or without Sarcopenia. Middle age was defined as between 45 and 65 years old, with elderly age over 65 years, and Sarcopenia was diagnosed by EWGSOP or AWGS (7, 16); (2) The study design was a randomized control trial (RCT); (3) Experimental groups received flavonoids or flavonoid-rich foods as interventions; (4) Control groups received a placebo supplement, and subjects taking exercise training are also taken into account; (5) The study provided available data to calculate average differences between baseline and endpoints, including skeletal muscle mass indicators, muscle strength indicators, and physical performance indicators.

Studies were eliminated if (1) the trial was conducted *in vitro* or in an animal model or if (2) the trial had a Non-RCT design, such as a case report, case series, or a prospectively designed trial without a comparison group.

### Data extraction and quality assessment

Relevant data were extracted including general characteristics of the study and population (authorship, year of publication, and type of study population), the number of cases and controls, type of intervention, intervention duration, and measured outcomes (skeletal muscle mass (SMM), skeletal muscle mass index (SMMI), appendicular skeletal muscle mass (ASM), appendicular skeletal muscle mass index (ASMI), lean body mass (LBM), gait speed, the Timed-Up and Go and 6-min walk distance) by 2 reviewers (YZL and YL) independently.

The quality of the included studies was assessed according to Cochrane Collaboration's tool for assessing the risk of bias, considering the following characteristics: (1) randomized sequence generation; (2) treatment allocation concealment; (3) blinding of participants; (4) completeness of the outcome data; (5) selective outcome reporting, and (6) other sources of bias (17). Within each domain, an independent judgment was made separately by the 2 reviewers (YZL and YL).

### Data synthesis and statistical analysis

The quality Statistical analysis was performed by RevMan (Review Manager, version 5.3; London, UK). Standard mean difference (SMD) along with the corresponding 95% confidence intervals (95% CIs) were used to examine the differences between the flavonoids and placebo groups. Estimates of statistical heterogeneity between the studies were described using Cochran Q (Chi-square test) and I^2^ statistics, where values of 25–49% were considered low, 50–74% considered moderate, and 75–100% considered high heterogeneity. A random-effects model was used if there was significant heterogeneity (*P* ≤ 0.05), and a fixed-effects model was used otherwise (18). To investigate whether the changes in outcomes were affected by the subjects' characteristics, subgroup analyses were conducted on whether participants were Sarcopenia or if they were intervened with exercise. Publication bias was evaluated by generating funnel plots and the Egger bias test, and a two-sided *p* value ≤ 0.05 was considered statistically significant, which was performed using Stata MP, version 15 (StataCorp; 2015; Stat Statistical Software: Release15 College Station, TX, USA).

The quality of the evidence and the robustness of the recommendations were evaluated using the GRADE (Grade of Recommendations Assessment, Development, and Evaluation) method. The quality of evidence was categorized as high, moderate, low or very low depending on the risk judgments of bias, inconsistency, vagueness, indivisibility and publication bias (19).

## Result

**Figure 1:**
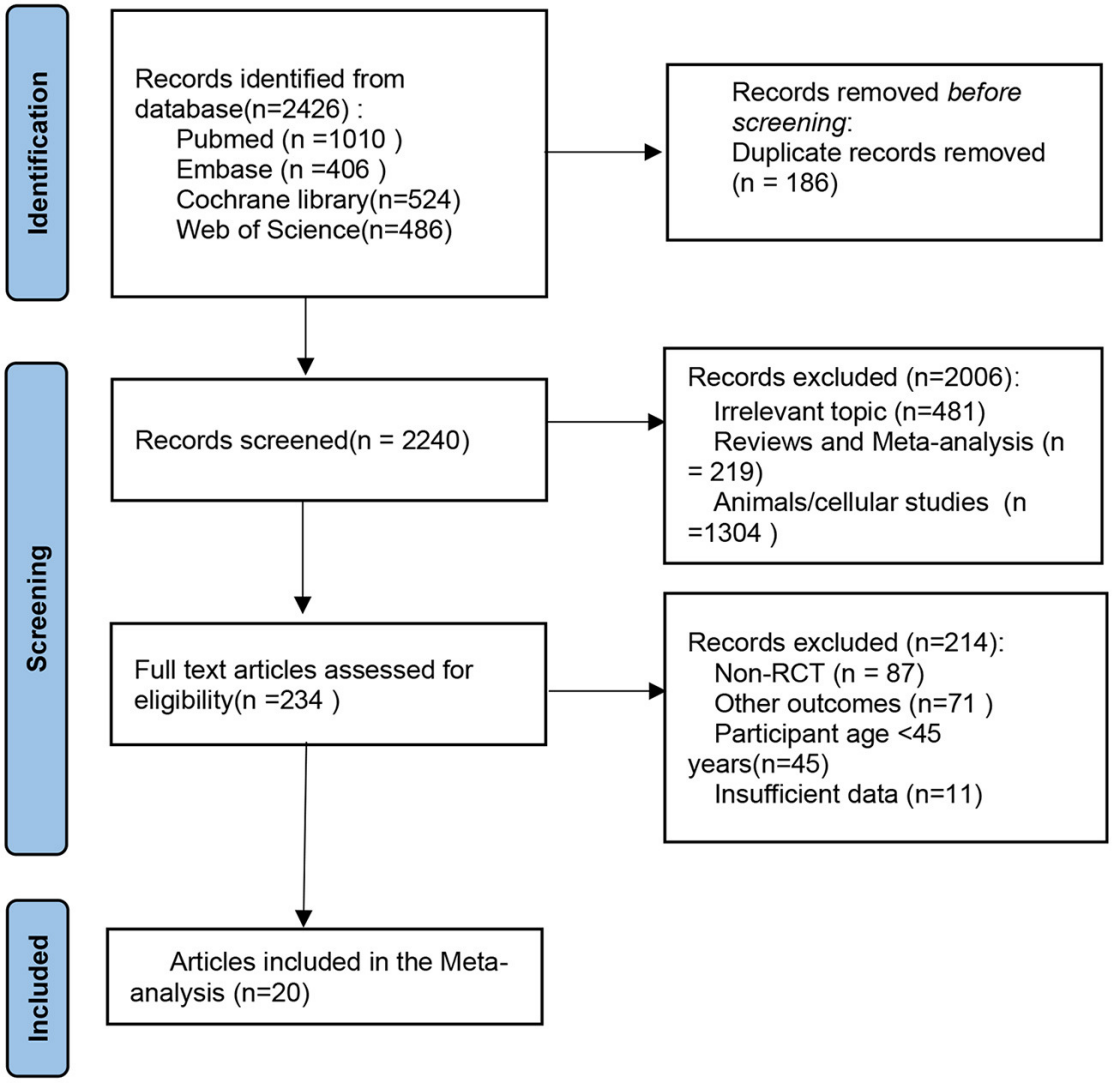
PRISMA flow diagram of the selection of the studies. PRISMA, Preferred Reporting Items for Systematic Reviews and Meta-Analyses.

The literature retrieval process is shown in Figure 1. A total of 2,426 articles were identified through the systematic search. After the removal of duplicates, 2,240 articles were screened for inclusion based on title and abstract, which resulted in 234 full-text articles considered for inclusion. After detailed reading, 214 articles were excluded for several reasons such as Non-RCTs, reporting irrelevant outcomes, including a population aged below 45 years, and the presence of insufficient data. In the end, 20 randomized placebo-controlled intervention studies were included in this systematic review, which enabled us to investigate the effects of flavonoids on skeletal muscle mass, strength, and physical performance (13, 20–38).

### Characteristics of the eligible studies

The included articles contain basic characteristics as shown in Table 1. Among them, six RCTs were conducted in Japan, four in Canada, four in Brazil, two in the USA, and one each in China, Mexico, Australia, and Iran. Fifteen studies used isoflavones or isoflavone-rich supplementations as intervention, two used catechin or epicatechin, and one used curcumin and rutin. The duration of the interventions spanned 8 to 24 weeks. Twelve studies conducted exercise training plans during supplement of flavonoids.

**Table 1:** Study characteristics of the included trials.

**References**	**Region**	**Study population**	**Age, years**	**Sex**	**Subjects, n**	**Duration**	**Type of Intervention**	**With Training**	**Outcomes**	**Conclusions**
Aubertin-Leheudre et al. (28)	Canada	Sarcopenic–obese postmenopausal women	50–70	F	12/6	24 weeks	Isoflavones 70 mg/d	No	LBM ↑ ASM ↑ ASMI ↑	The isoflavone supplementation increased significantly fat-free mass, appendicular fat-free mass, and appendicular fat-free mass index.
Maesta et al. (29)	Brazil	Postmenopausal women	45–70	F	14/11	16 weeks	Isoflavones 50 mg/d	Resistance training	SMM ↔	Soy protein supplementation did not influence the indicators of body composition. The increase in muscle mass was correlated with resistance training.
Choquette et al. (38)	Canada	Overweight-to-obese postmenopausal women	50–70	F	10/12	6 months	Isoflavones 70 mg/d	No	LBM ↔ SMMI ↔ Grip strength ↔	Exercise training could improve muscle tissue strength, function, and quality. Isoflavones, irrespective of exercise, did not produce changes in these variables.
Kim et al. (24)	Japan	Sarcopenic women	≥75	F	32/32	3 months	Catechin 540 mg/d	Multicomponent exercise	SMM ↑ ASM ↑ Grip strength ↔ Gait speed ↑ TUG ↓	The combination of exercise and tea catechin effectively enhanced muscle mass and walking ability, but the changes in muscle strength were not significant.
Lebon et al. (30)	Canada	Postmenopausal women	50–70	F	19/15	6 months	Isoflavones 70 mg/d	Aerobic and resistance training	SMMI ↑	The combination of isoflavones and a mixed 6-month exercise program had a significant effect on body composition, by increasing muscle mass index.
Beavers et al. (36)	USA	Abdominally obese	60–79	F, M	12/12	12 weeks	Isoflavones 60–135 mg/d	No	LBM ↓ Grip strength ↔	In soy protein-based meal replacements combined with groups, despite significant reductions in lean mass, no significant changes.
Maltais et al. (20)	Canada	Sarcopenia elderly men	60–75	M	8/10	4 months	Soy beverage	Resistance training	SMM ↔ SMMI ↔ ASMI ↔ Gait speed ↔ TUG ↔	Resistance training is an effective way to increase muscle mass and physical capacity, regardless of soy beverage-riched essential amino acid supplements.
Thomson et al. (35)	Australia	Healthy	50–79	F, M	26/23	12 weeks	Soy protein 27 g/d	Resistance training	LBM ↔ Grip Strength ↔ 6-min walk distance ↔	Lean mass, handgrip strength, and the 6-min walk test increased significantly, with no difference between diets.
Kinoshita et al. (26)	Japan	Knee osteoarthritis	54–90	F, M	25/23	16 weeks	Licorice Flavonoid Oil 300 mg/d	No	SMM ↑	Licorice Flavonoid Oil supplementation has effects on increasing muscle mass of elderly populations, especially in the body trunk.
Orsatti et al. (22)	Brazil	Postmenopausal Women	≥45	F	16/16	16 weeks	Isoflavones 50 mg /d	Resistance training	SMM ↔	Adding isoflavone-enriched soy to milk resulted in a greater increase in muscle strength but not in muscle gain after resistance training in healthy participants.
Mafi et al. (31)	Iran	Sarcopenic older subjects	65–75	M	15/14	8 weeks	Epicatechin 1 mg/kg/d	Resistance training	ASMI ↑ TUG ↓	ASMI and TUG enhanced significantly in all experimental groups, especially in resistance training with epicatechin group.
Barbosa et al. (27)	Brazil	Postmenopausal women	50–70	M	16/14	10 weeks	Isoflavones 100 mg/d	Aerobic and resistance exercise	LBM ↔ 6-min walk distance ↑	No differences were found for fat-free mass in isoflavone group. In addition, isoflavone group increased the performance in walk test.
Imaoka et al. (34)	Japan	Older Subjects	≥60	F, M	37/37	90 days	Soy peptide 4 g/d	Multicomponent exercise	ASMI ↑ Grip strength ↑ Gait speed ↑	A combination of exercise and soy peptide supplementation was effective in improving gait speed, grip strength, and ASMI in elderly adults.
Munguia et al. (23)	Mexico	Older Subjects	55–70	F, M	20/20	12 weeks	Flavonoid-rich natural cocoa beverage (179 mg)	No	SMMI ↑ Grip strength ↑ 6-min walk distance ↑ TUG ↓	Regular flavonoids consumption positively affects performance on the Up and Go test, skeletal muscle index, and the handgrip strength in the elderly people.
Boutry-Regard et al. (13)	Japan	Older Subjects	≥65	F, M	10/15	12 weeks	Curcumin and rutin 500 mg/d	No	LBM ↔ Gait speed ↑	Curcumin and rutin-enriched supplementations improved gait speed significantly, but did not have any effect on total lean mass.
McDermott et al. (37)	USA	Peripheral artery disease	≥60	F, M	22/21	6 months	Epicatechin 75 mg/d	No	6-min walk distance ↑	Flavanol-rich cocoa improved walking performance significantly in people with lower extremity peripheral artery disease.
Kinoshita et al. (32)	Japan	Middle-aged and older subjects	≥40	M	32/32	16 weeks	Licorice flavonoid oil 300 mg	Resistance training	SMM ↔ Grip strength ↔	Muscle mass and handgrip strength did not show significant increase in Licorice flavonoid oil group.
Roschel et al. (25)	Brazil	Pre-frail and frail elderly.	≥65	F, M	22/22	16 weeks	Soy 30 g/d	Resistance training	LBM ↔ ASM ↔ Grip strength ↔ TUG ↔	Supplementation with soy failed to enhance resistance-training effects on appendicular lean mass, handgrip strength and timed-up-and-go.
Chunlei et al. (33)	China	Sarcopenic older subjects	≥65	F, M	31/30	6 months	Soy protein 17.6 g/d	No	ASMI ↔ Grip strength ↔ Gait speed ↔	Supplementation soy, or whey-soy blended protein equally maintained lean muscle mass and physical performance in older adults with low lean mass. There were no significant differences among treatment groups.
Yasunobu et al. (21)	Japan	Healthy	≥65	F, M	27/25	24 weeks	Catechins 540 mg/d	Resistance training	SMMI ↔ Grip strength ↔ Gait speed ↔	There were no between-group differences in the percent change from pre- to the post-intervention of Catechins in the SMI, grip strength, and gait speed.

SMM, skeletal muscle mass; SMMI, skeletal muscle mass index; ASM, appendicular skeletal muscle mass; ASMI, appendicular skeletal muscle mass index; LBM, lean body mass; TUG, Timed-Up and Go test;↑ Increased, ↓ Decreased, ↔ Maintained.

### Assessment of risk of bias

**Figure 2:**
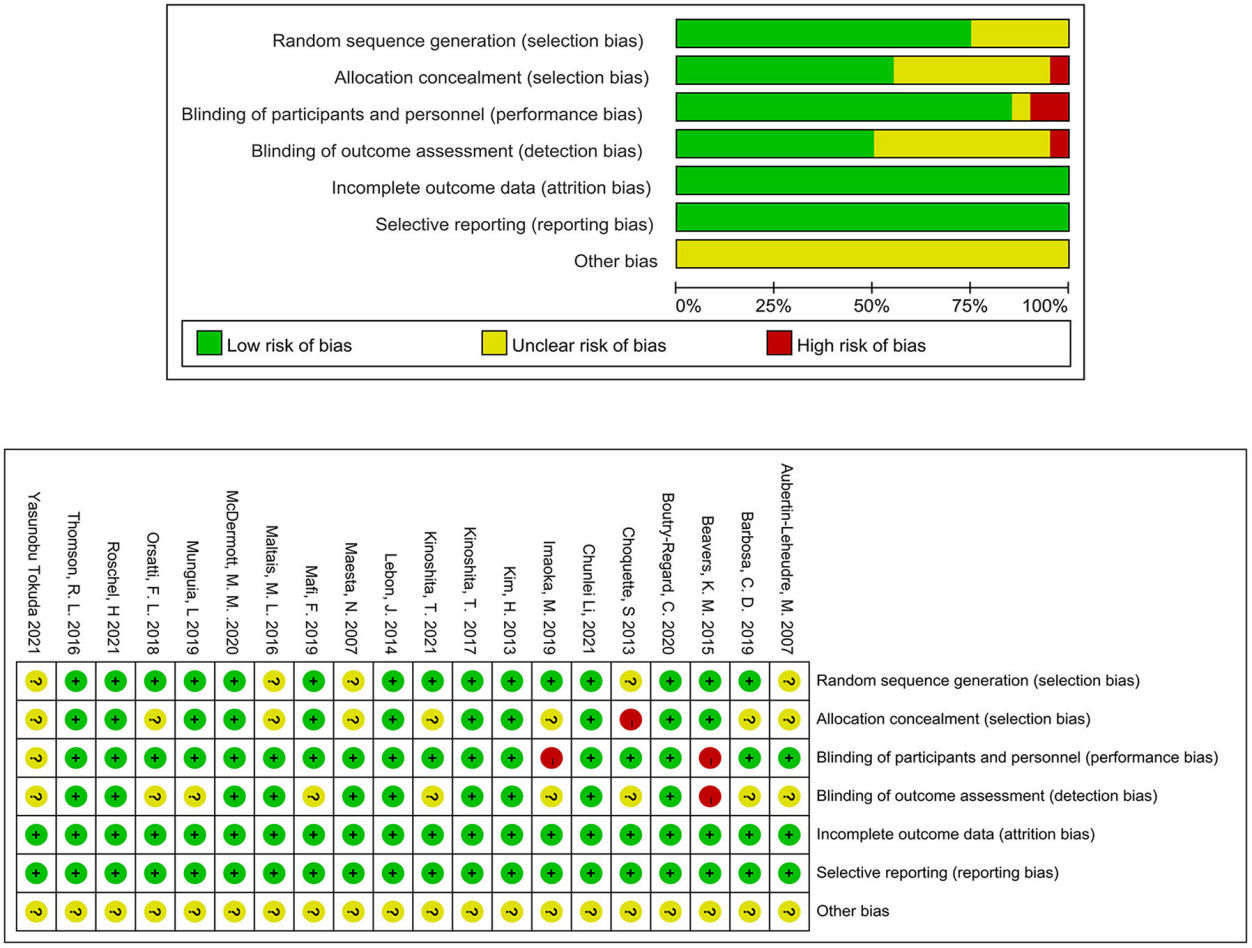
Risk of bias for all included studies.

The studies' risks of bias are shown in Figure 2. They were typically at low risk of bias for most domains including completeness of the outcome (100%), selective reporting (100%), and random sequence generation (75%) and at an unclear risk of bias for the other bias (100%). The allocation concealment was at low risk in eleven studies (55%), unclear in eight studies (40%), and at high risk in one study (5%). The blinding of the participants and personnel was at low risk in ten studies (50%), unclear in eight studies (40%), and at high risk in two studies (10%). The blinding of the outcome assessment was at low risk in ten studies (50%), unclear in nine studies (45%), and high risk in one study (5%).

### The effects of flavonoids on muscle mass indicators

Of the eighteen studies that determined the effects of flavonoids on muscle mass outcomes, Results on muscle mass outcomes were clustered based on the following domains evaluated in the studies, (i) SMM or SMMI, (ii) ASM or ASMI, (iii) LBM.

#### Skeletal muscle mass

**Figure 3:**
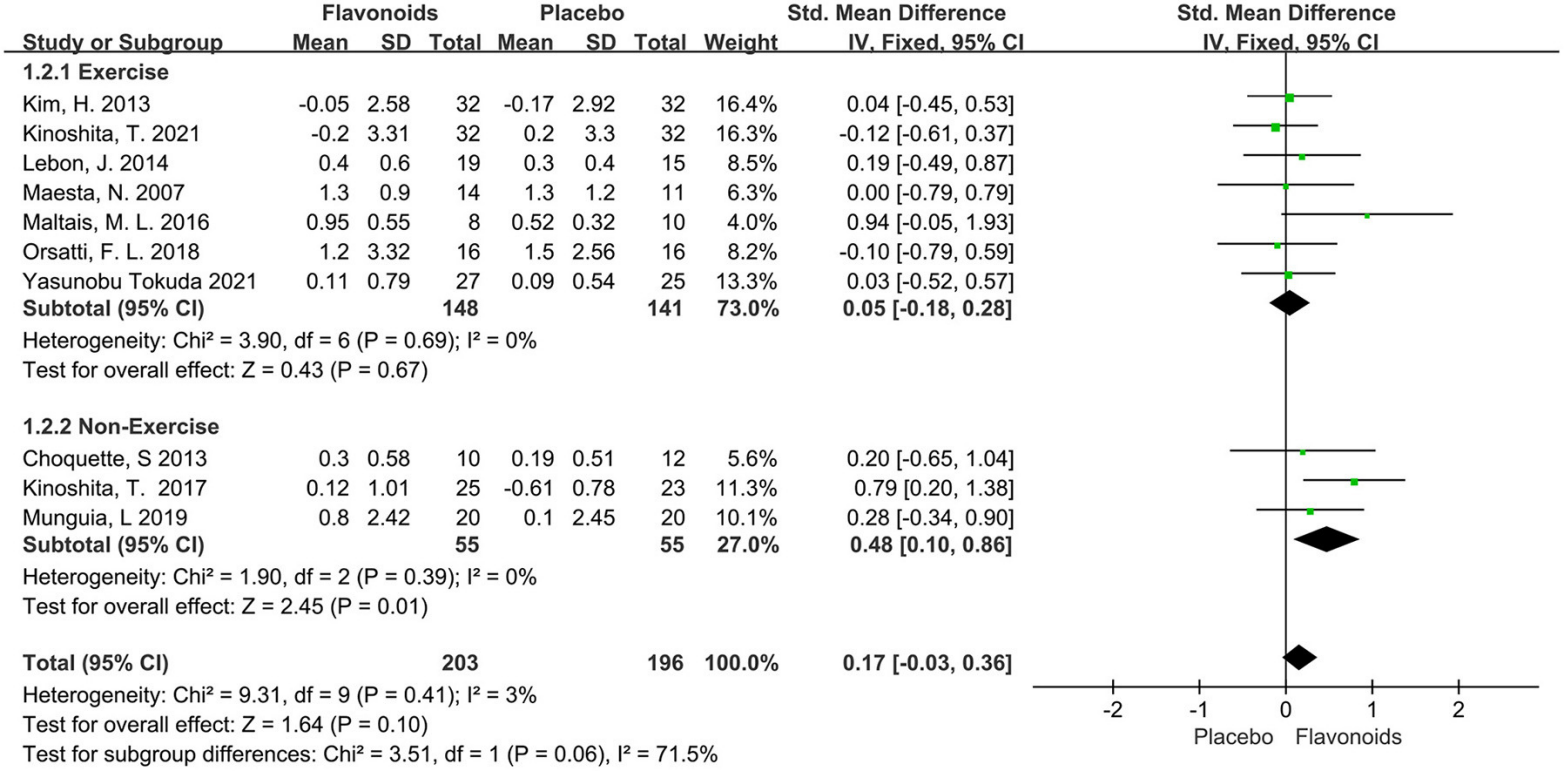
Forest plots of the included studies assessing effects of flavonoid supplementation on skeletal muscle mass categorized by exercise training intervention. IV, inverse-variance method; Fixed, fixed effect.

A total of 10 trials with 399 participants reported the effects of flavonoids on SMM or SMMI. The pooled effect showed that supplementation with flavonoids did not exert muscle mass change (SMD = 0.17; 95% CI: −0.03, 0.36; *P* = 0.10), with an insignificant between-study heterogeneity (I^2^ = 3%, *P* = 0.41). A subgroup analysis was performed to explore differences in muscular change according to the combination with exercise intervention. We found that flavonoids for participants without taking exercise had a significant increase in SMM or SMMI compared to the control group (SMD = 0.48; 95% CI: 0.10, 0.86; *P* = 0.01) (Figure 3).

#### Appendicular skeletal muscle mass

**Figure 4:**
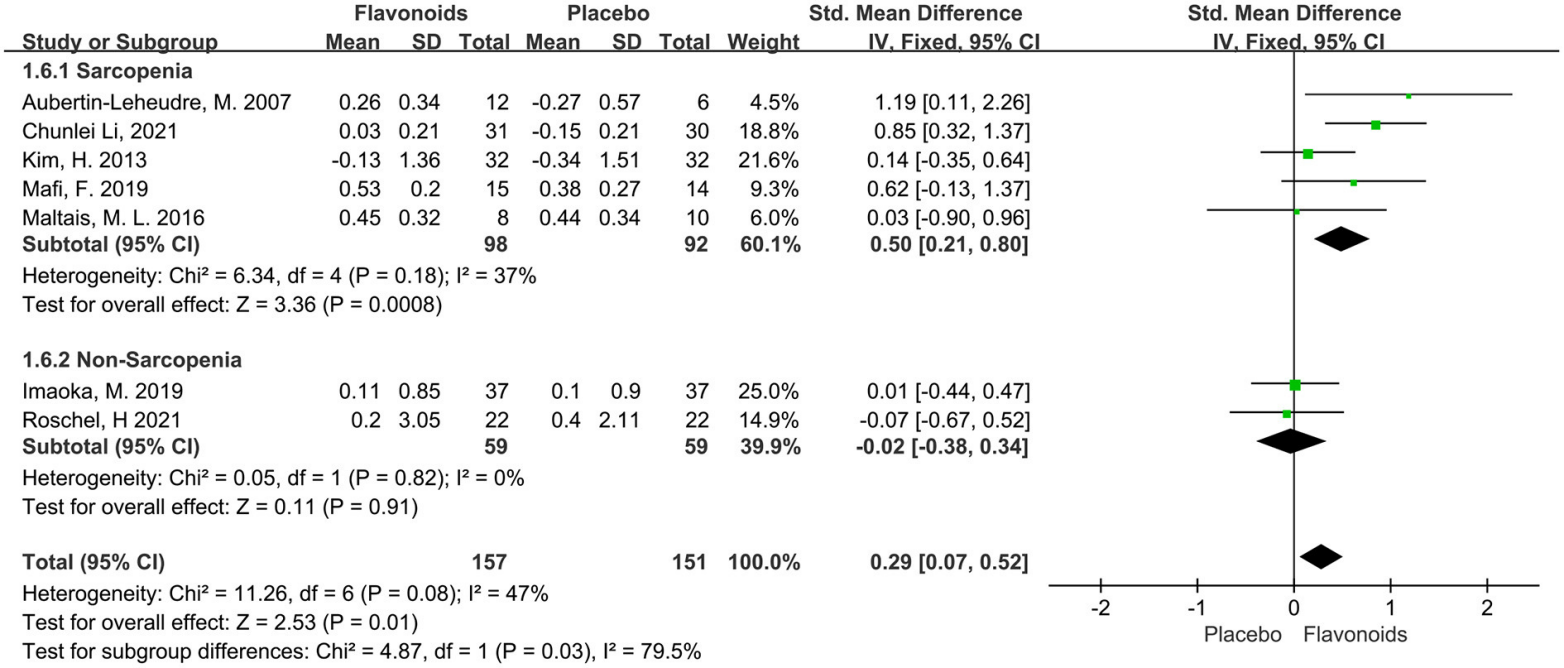
Forest plots of the included studies assessing effects of flavonoid supplementation on appendicular muscle mass categorized by Sarcopenia. IV, inverse-variance method; Fixed, fixed effect.

Seven studies including a total of 308 participants reported ASM or ASMI as an outcome measure. The combined results showed marginal significant benefits on the ASM or ASMI following flavonoid consumption (SMD = 0.29; 95% CI: 0.07, 0.52; *P* = 0.01), with low heterogeneity among the studies (I^2^ = 47%, *P* = 0.08). When the subgroup analysis was based on Sarcopenia, we found that studies for Sarcopenia showed significant improvements in the ASM or ASMI of subjects who received flavonoids (SMD = 0.50; 95% CI: 0.21, 0.80; *P* < 0.01), while those without Sarcopenia failed to show significant differences in the ASM or ASMI (Figure 4).

#### Lean body mass

**Figure 5:**
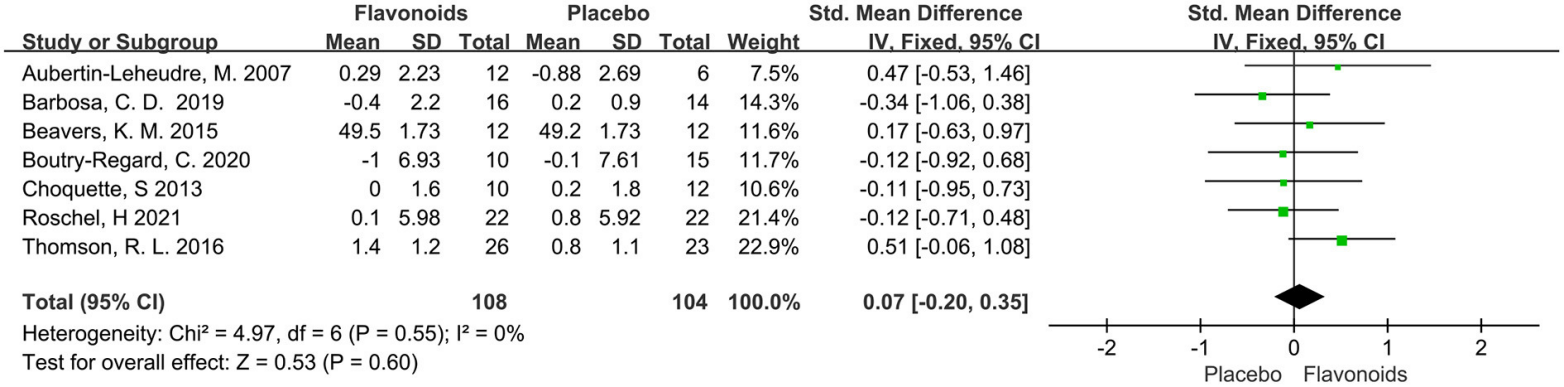
Forest plots of the included studies assessing effects of flavonoid supplementation on lean body mass. IV, inverse-variance method; Fixed, fixed effect.

A total of 7 trials with 212 participants provided available data on the LBM, and the summary estimate showed that flavonoids supplementation did not exert significant effect on LBM (SMD = 0.07; 95% CI: −0.20, 0.35; *P* = 0.60) with no heterogeneity across the studies (I^2^ = 0.0%, *P* = 0.55) (Figure 5).

### The effects of flavonoids on muscle strength

**Figure 6:**
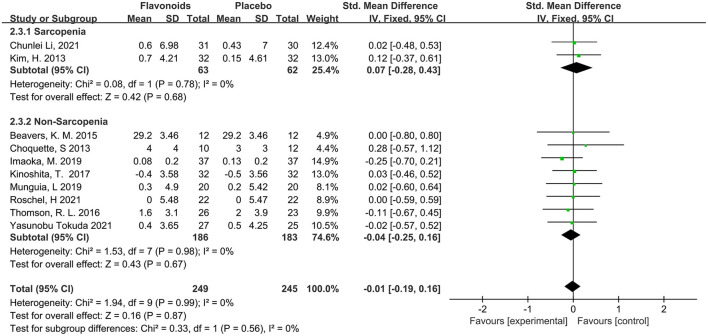
Forest plots of the included studies assessing effects of flavonoid supplementation on grip strength categorized by Sarcopenia. IV, inverse-variance method. Fixed, fixed effect.

This systematic analysis makes grip strength a representative parameter of muscle strength. A total of 10 studies with 494 participants reported the measures of grip strength, no difference was observed in the effect of flavonoids supplementation on grip strength compared with the control condition (SMD = −0.01; 95% CI: −0.19, 0.16; *P* = 0.87) with no intra-studies heterogeneity (I^2^ = 0%, *P* = 0.99). When the subjects were stratified in the combination with Sarcopenia, the results showed no difference in grip strength (SMD = 0.07; 95% CI: −0.28, 0.43; *P* = 0.68), with no intra-subgroup heterogeneity (I^2^ = 0.0%, *P* = 0.56) (Figure 6).

### The effects of flavonoids on physical performance

Results on physical performance outcomes were clustered based on the following domains evaluated in the studies, (i) Gait speed, (ii) Timed-Up and Go test, and (iii) 6-min walk distance.

#### Gait speed

**Figure 7:**
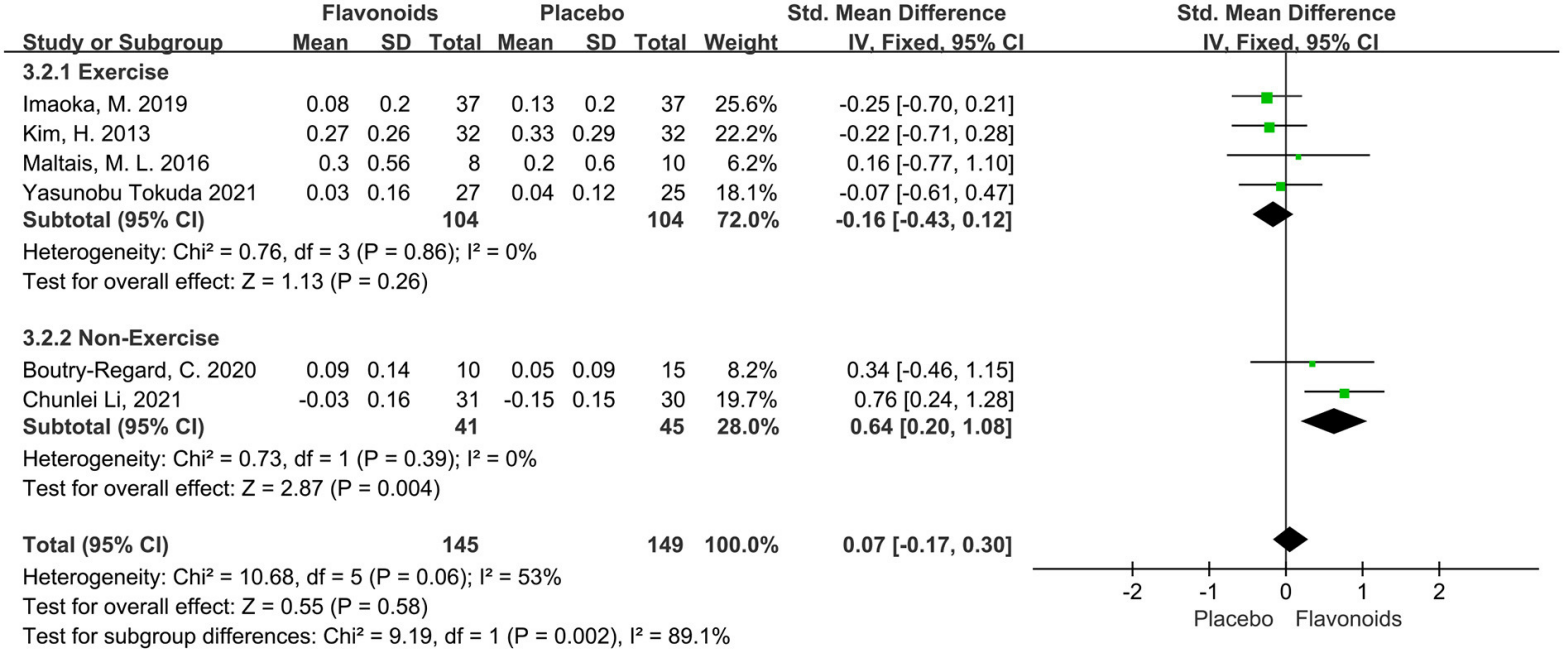
Forest plots of the included studies assessing effects of flavonoid supplementation on gait speed categorized by exercise intervention. IV, inverse-variance method; Fixed, fixed effect.

Six studies including a total of 294 participants reported gait speed as an outcome measure. Combined results from the fixed-effects model showed no significant impact on the gait speed following flavonoid consumption (SMD = 0.07; 95% CI: −0.17, 0.30; *P* = 0.58), with moderate heterogeneity among the studies (I^2^ = 53%, *P* = 0.06). Stratified analysis conducted according to combination with exercise intervention showed that flavonoids had a significant beneficial effect on gait speed for the non-exercise group (SMD = 0.64; 95% CI: 0.20, 1.08; *P* < 0.01) (Figure 7).

#### Timed up and go test

**Figure 8:**
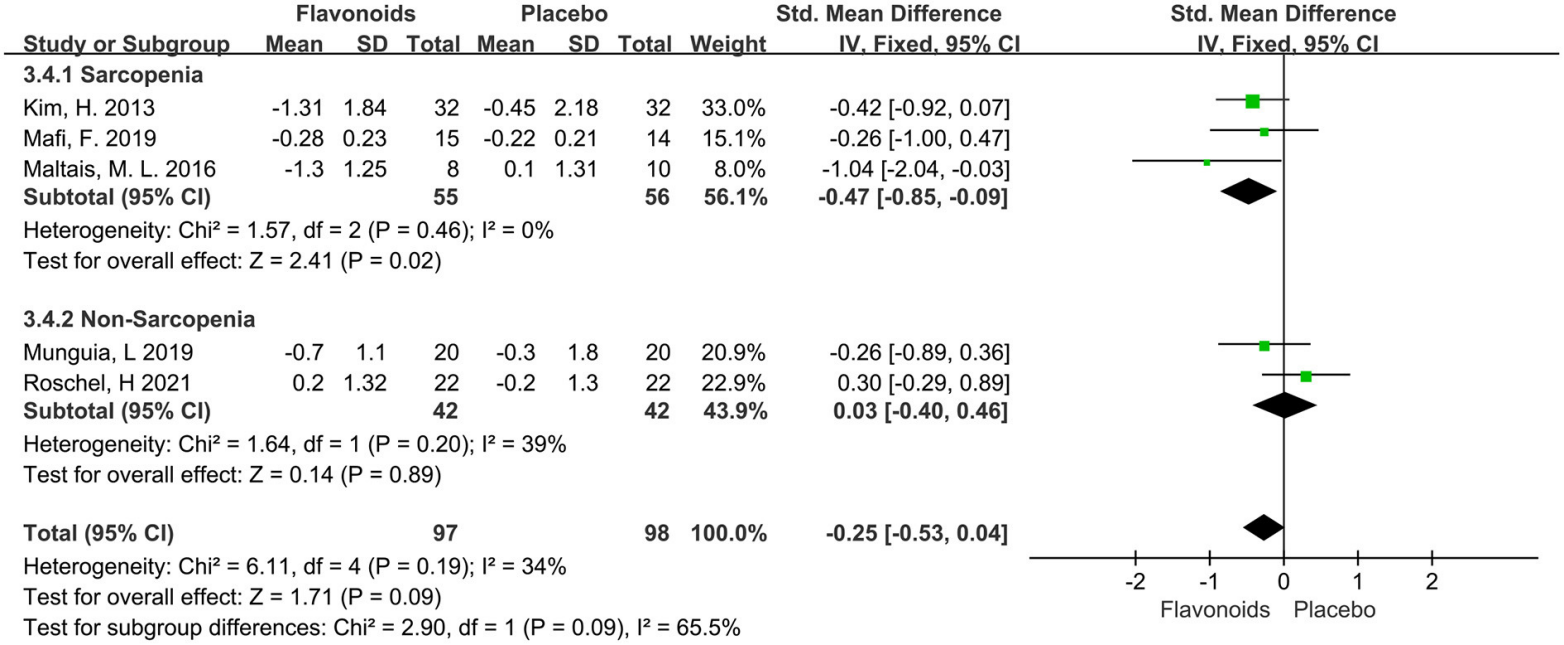
Forest plots of the included studies assessing effects of flavonoid supplementation on Timed-Up and Go test categorized by Sarcopenia. IV, inverse-variance method. Fixed, fixed effect.

The variation of Timed-Up and Go test was reported in 5 trials of total 197 participants, and no significant effect was observed (SMD = −0.25; 95% CI: −0.53, 0.04; *P* = 0.09), with low heterogeneity (I^2^ = 34%, *P* = 0.19). The trials stratified by Sarcopenia indicated that the pooled effect of flavonoid supplements significantly reduced the Timed-Up and Go for the Sarcopenia population (SMD = −0.47; 95% CI: −0.85, −0.09; *P* = 0.02) (Figure 8).

#### 6-min walk distance

**Figure 9:**
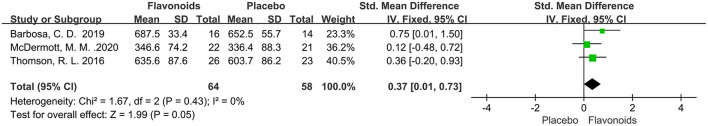
Forest plots of the included studies assessing effects of flavonoid supplementation on 6-min walk distance. IV, inverse-variance method; Fixed, fixed effect.

Three trials of total 122 participants reported the effect of flavonoids supplementation on 6-min walk distance. The pooled effect showed that flavonoids supplementation has significant benefits effect on 6-min walk distance (SMD = 0.37; 95% CI: 0.01, 0.73; *P* = 0.05), with no heterogeneity across studies (I^2^ = 0%, *P* = 0.43) (Figure 9).

### Sensitivity analysis

**Figure 10:**
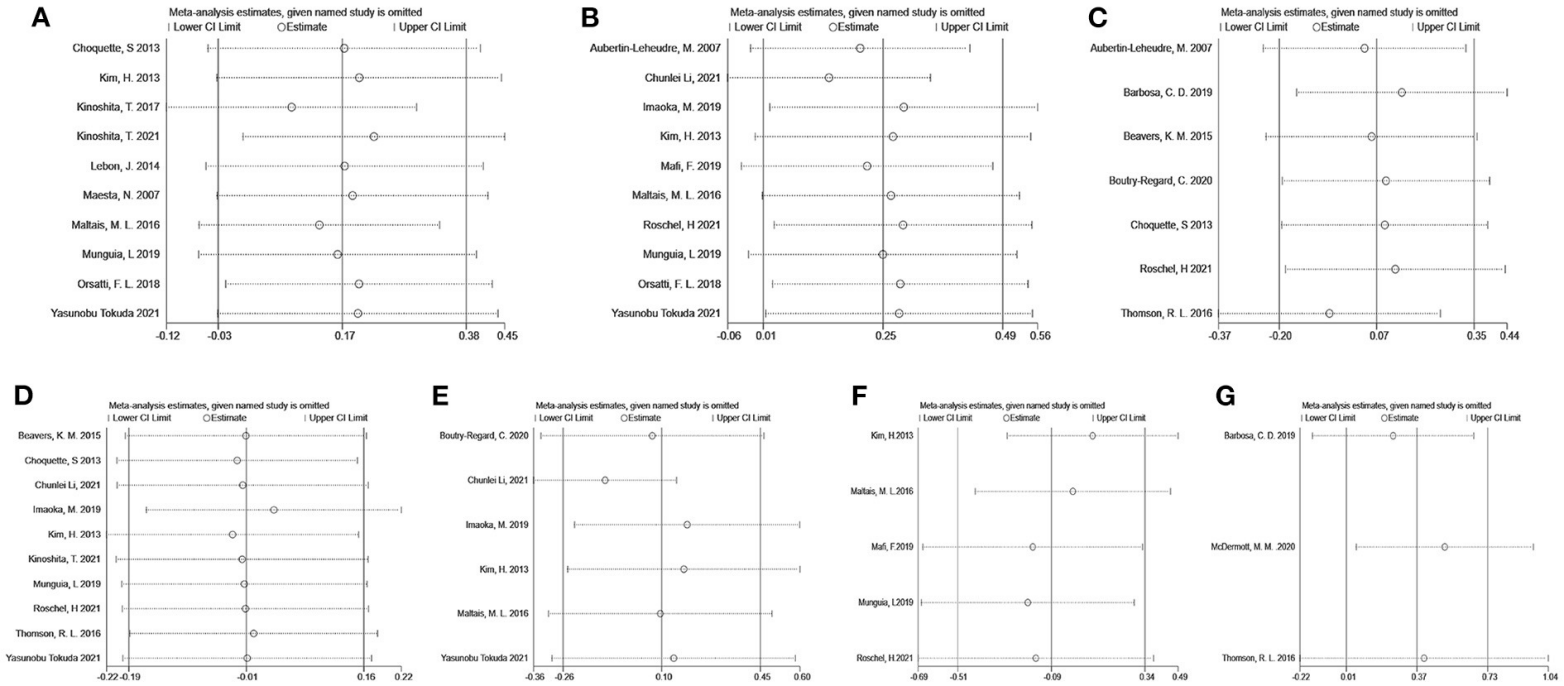
Sensitivity analyses by skeletal muscle mass **(A)**, appendicular muscle mass **(B)**, lean body mass **(C)**, grip strength **(D)**, gait speed **(E)**, Timed-Up and Go test **(F)**, and 6-min walk distance **(G)**.

Sensitivity analysis to appraise the stability were carried out using the leave-one-out approach (Figure 10). It revealed that the effect of flavonoids on SMM, ASM, LBM, Grip strength, Gait speed, Time-Up and Go test, and 6-min walk distance was not substantially changed by excluding a particular study. Taken together, these results indicated that this meta-analysis showed good reliability.

### Publication bias and evidence certainty

**Figure 11:**
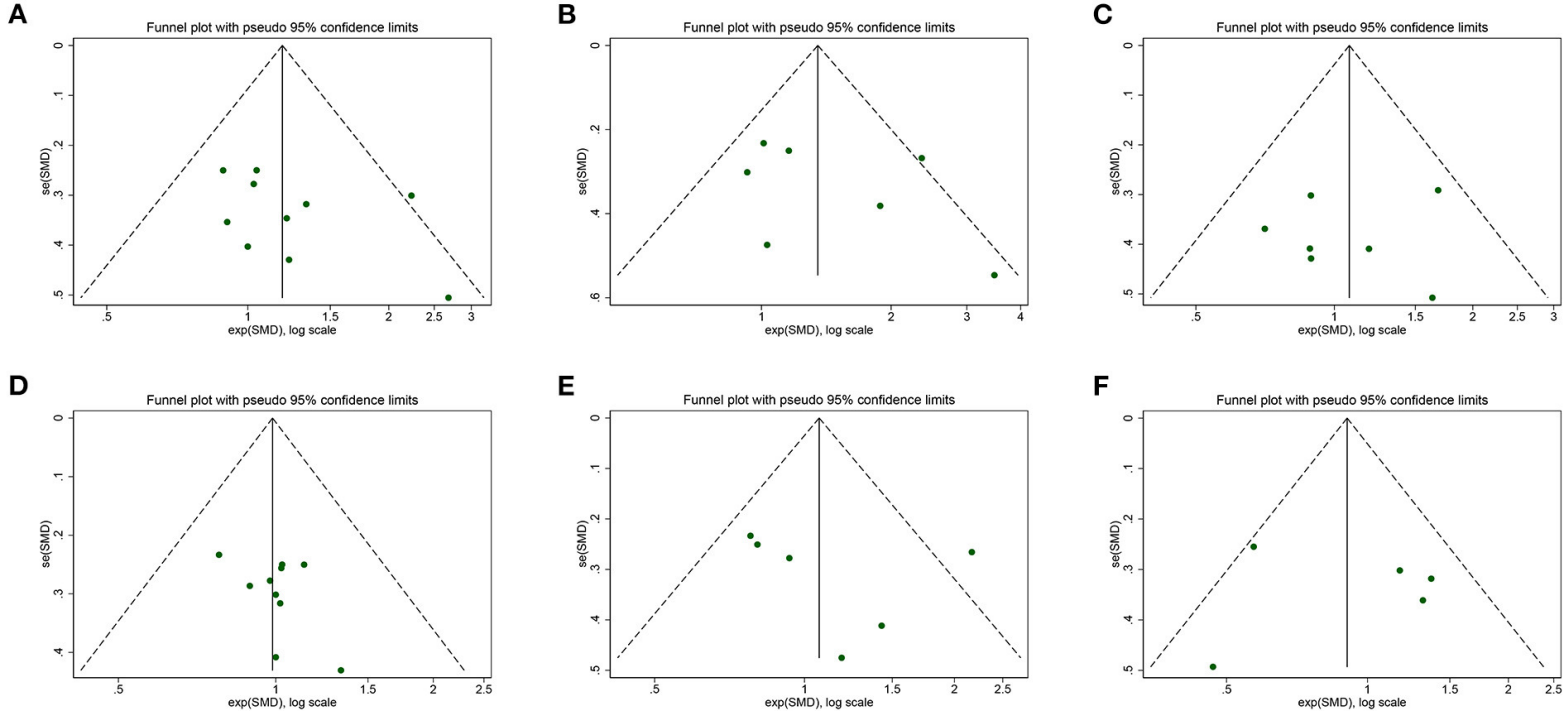
Funnel plot of meta-analysis result: **(A)** skeletal muscle mass, **(B)** appendicular muscle mass, **(C)** lean body mass, **(D)** grip strength, **(E)** gait speed, **(F)** Timed-Up and Go test.

The visual inspection of funnel plots revealed an asymmetric distribution in SMM, ASM, and Gait speed, indicating potential publication bias (Figure 11). Therefore, we further performed the Egger bias test for quantitative detection. The Egger's test showed no significant publication bias regarding SMM (*t* = 1.30, *P* = 0.230), ASM (*t* = 0.97, *P* = 0.378), LBM (*t* = −0.26, *P* = 0.805), Grip strength (*t* = 1.46, *P* = 0.184), Gait speed (*t* = 0.64, *P* = 0.555), Time-Up and Go test (*t* = 0.06, *P* = 0.952) and 6-min walk distance (*t* = 1.32, *P* = 0.413). The evidence certainty for measured outcomes was rated from moderate to very low according to GRADE, with a summary of the effects of the flavonoids on muscle mass, strength, and physical performance summarized in Table 2.

**Table 2:** Summary effects of flavonoids on the outcomes of interest among the included studies, publication bias, and quality evidence of the Grading of Recommendations Assessment, Development and Evaluation (GRADE).

**Outcomes**	**No. of studies**	**No. of participants**	**Effect estimate**	***p*-value**	**Heterogeneity (I^2^)**	**Publication bias**	**Certainty of the evidence (GRADE)**
Skeletal muscle mass	10	399	0.17(−0.03, 0.36)	0.10	3%	0.230	⊕⊕○○ LOW
Appendicular muscle mass	7	308	0.29(0.07, 0.52)	0.01	47%	0.378	⊕⊕⊕○ MODERATE
Lean body mass	7	212	0.07(−0.20, 0.35)	0.60	0%	0.805	⊕○○○ VERY LOW
Grip strength	10	494	−0.01(−0.19, −0.16)	0.87	0%	0.184	⊕⊕⊕○ MODERATE
Gait speed	6	294	0.07(−0.17, 0.30)	0.58	53%	0.555	⊕⊕○○ LOW
Time-Up and Go test	5	197	−0.25(−0.53, 0.04)	0.09	34%	0.413	⊕○○○ VERY LOW
6-min walk distance	3	122	0.37(0.01, 0.73)	0.05	0%	0.952	⊕⊕⊕○ MODERATE

## Discussion

This meta-analysis with 20 RCTs involving 796 elderly participants showed that flavonoid supplementation was associated with an increase in appendicular muscle mass by 0.29 kg for the middle-aged and elderly, especially for the Sarcopenia population. In terms of muscle strength, we found that flavonoid supplementation did not elicit greater handgrip strength. For muscle performance, flavonoid administration enhanced performance in the 6-min walk distance and facilitated sarcopenia up-and-go time.

Our result suggests that flavonoid supplementation has a beneficial effect on appendicular muscle mass which is recognized to be responsible for locomotion and physical independence, similar to a recently published systematic review and meta-analysis indicating that flavonoid improved muscle cross-sectional area and muscle mass (39). Muscle fiber loss and muscle fiber atrophy are the main mechanisms of muscle deterioration with aging. The former is primarily due to imbalances between muscle protein synthesis (MPS) and muscle protein breakdown (MPB). It has been observed that the blunting of MPS in combination with the suppressed inhibition of MPB exacerbates muscle fiber loss in the elderly (40). Multiple pathways participate in such skeletal muscle protein turnover at the molecular level. There is evidence that flavonol glycoside has skeletal muscle growth and differentiation effects by promoting the PI3K/Akt/mTOR pathways, which is the key molecule to promote protein synthesis and block protein degradation, and it can also down-regulate Smad pathways, which are negative regulators of skeletal muscle growth (41). Another study reported that licorice flavonoid oil promoted the phosphorylation of mTOR and p70S6K, which are involved in muscle synthesis pathways (42). Meador et al. (43) found that the green tea polyphenol epigallocatechin-3-gallate reduced the atrogin-1 and MuRF-1 in aged rats, the biomarker of muscle atrophy. Munguia et al. (44) found that epicatechin attenuated muscle damage by reducing the expression of Foxo1 in aged mice, a transcription factor of ubiquitin-proteasome for proteolytic activation. Chang and his colleagues also found that epigallocatechin gallate attenuated muscle atrophy and protein degradation upregulated miR-486-5p in aged mice, a microRNA modulates skeletal muscle epigenetic expression (45). Meanwhile, insulin, with a similar structure to IGF-1, potentially activates the anabolic mTOR pathway and inhibits the catabolic ubiquitin-proteasome pathway, contributing to maintaining skeletal muscle protein balance (46). Such balance can be broken because of the resistance to insulin in age-related Sarcopenia. Guevara et al. observed that genistein, a subclass of isoflavones, could improve insulin resistance and increase skeletal muscle fatty acid oxidation through changing gut microbiota (47). Bowser et al. (48) investigated the insulin-enhancing effects and insulin-mimetic activities of cocoa procyanidins in human skeletal muscle metabolism, which may lead to inducting MPS and contribute to the inhibition of MPB. Apart from MPS and MPB, recent studies demonstrated that anabolic resistance is the major driver of age-related muscle loss (40). Anabolic resistance is a situation where the skeletal muscle is unable to respond appropriately to these anabolic stimuli (e.g., nutrition and exercise) by stimulating protein synthesis (49–51). The main reason for this blunted response is fat infiltration into skeletal muscle and muscle ectopic fat deposition (52). Ohmae et al. (53) found that quercetin attenuated adipogenesis and fibrosis in human skeletal muscle. Xi et al. (54) found that baicalin attenuates insulin resistance and ectopic fat storage in skeletal muscle. A previous systemic review has shown that flavonoid-enriched diets may benefit lipid metabolism (55), indicating that flavonoids may improve anabolic resistance by improving lipid metabolism and ectopic deposition in muscle. On the other hand, anabolic resistance means that a more intense exercise is required to allow elderly subjects to gain muscle mass and enhance muscle strength. However, with weak sports ability and without the supervision of professionals, there are certain risks in sports reinforcement for the elderly. Therefore, exercise strengthening is not suitable for all the elderly. In our subgroup analysis, we found that non-exercise people can have better muscle mass benefits after supplementing flavonoids. Isoflavones have been usually used as a surrogate for hormone replacement therapy (HRT) for its plant estrogens property. A 12-month clinical trial combining exercise and HRT showed that it was of benefit on body composition only to non-exercisers (56), which is consistent with our present result. LBM consists of muscles, organs, and bones. Although a previous systematic review and meta-analysis has shown that soy isoflavones have the effect of increasing bone mineral density (57), our results did not observe beneficial effects on LBM. The sample size and intervention time might have contributed to the inconsistency in the findings between studies.

With regard to muscle strength, the data analyzed do not allow us to draw any conclusions about the potential effect of flavonoids on handgrip in middle-aged adults and seniors. Our results are similar to previous systematic reviews that supplementation with dairy protein, well-established countermeasures against Sarcopenia, did not improve grip strength in older adults, with or without Sarcopenia (58). Given that muscle strength decreases at a faster rate compared with muscle mass and has better efficacy in predicting adverse outcomes, EWGSOP2 elevates low muscle strength to the forefront as a primary indicator of probable Sarcopenia (7). Actually, muscle strength does not depend solely on muscle mass, and the correlations between muscle strength and muscle mass are inconsistent and not linear (24). Dynapenia, defined as the loss of muscle strength and power, is specifically proposed to differentiate Sarcopenia (59). The evidence convincingly indicates that with age, muscle fibers undergo continued denervation and reinnervation owing to the accelerated loss of motor neurons in the spinal cord (60). Such a process is probably one of the main contributors to muscle atrophy resulting in the loss of muscle strength. Choi et al. found that dietary apigenin of flavone inhibits denervation-induced muscle atrophy by down-regulating the expression of inflammatory cytokines [tumor necrosis factor-α (TNF-α), interleukin-6 (IL-6)] (61). Tabata et al. (62) also found that isoflavone aglycone could modulate muscle atrophy after denervation in mice based on the modulation of apoptosis-dependent signaling. It seems that the anti-denervation effect of flavonoids is theoretically inconsistent with our negative findings in muscle strength. However, age-related muscle fiber type conversion should be taken into account. Aging can cause remarkable shrinkage of explosive fast-twitch type II fibers and promote their transformation into slow-twitch type I fibers (63). Therefore, the loss in muscle strength associated with Sarcopenia might also be a consequence of the preferential atrophy of type II fibers, responsible for higher intensity (64). Chen et al. found that quercetin could promote skeletal fiber to switch from glycolytic type II to oxidative type I through Adiponectin signaling pathway (65). Xue et al. (66) also found that naringin induced skeletal muscle fiber type transformation from II to type I by activating the AMPK/PGC-1α signaling pathway, which supports the notion that flavonoids could not improve muscle strength due to their muscle fiber switch property. Although flavonoids decrease Type II fibers leading to a reduction in power bursts, they have been shown to improve the endurance of activity which we will discuss below.

In order to evaluate physic performance, the included studies evaluated the subjects' gait speed, Timed-Up and Go, and 6-min walk distance. Our research has found that flavonoid supplementation could increase gait speed and 6-min walk distance. Physical performance is a multidimensional concept that goes beyond skeletal muscle, and it also involves many other body organs and systems (cardiovascular aspects, peripheral nervous and vascular function, motivation, balance…) (67), leading to falls, fractures, and even death directly in aging. In 2018, EWGSOP2 has purposed physical performance to categorize the severity of Sarcopenia (7). Gait speed and 6-min walk distance are diagnostic indicators of Sarcopenia with limited mobility (68). The latter is a commonly used test for the objective assessment of cardiovascular disease, pulmonary disease, frailty, and cancer, which is also predictive of morbidity and mortality in patients with chronic obstructive pulmonary diseases or congestive heart failure (69). Our results show that flavonoids provide a statistically significant benefit in 6-min walk distance. One reason perhaps is their well-documented cardioprotective effects. Several studies confirmed that flavonoids may exert their cardiovascular protective properties through various signaling pathways, such as AMPK, PPAR-γ, PGC-1α, and NF-κB (70, 71). Besides the contribution of the cardiopulmonary function to physical performance, the peripheral vascular system in skeletal muscle is crucial to maintaining physical activity. Older subjects have impairments in the vascular structure and function, including reductions in leg blood flow (72), decrements in microvascular function (73), loss of endothelial cells, and a reduced muscle capillary density (74). These changes have the potential to further compromise physical performance by affecting the delivery of energy, nutrients, and oxygen. Sian et al. found that the cocoa favanols condition displayed significant increases in the lower limb muscles microvascular blood volume (MBV), thus enhancing nutrient and oxygen delivery (75). Roberts et al. (76) found that catechin-rich green tea promoted peripheral vascular function by flow-mediated vascular dilation of upper and lower limbs. Heiss et al. (77) found that cocoa flavanols increased circulating nitric oxide (NO) with enhanced vasodilation and improved muscle perfusion, which could be reversed by NO-synthase inhibitor L-NAME. Similarly, Ludovici et al. summarized the vascular protective effects of cocoa flavanols in their reviews (78). Accompanied by the deterioration of the vascular system, the oxygen delivery and utility are restricted, which are the core steps for aerobic capacity. Yeh et al. (79) found that pueraria isoflavone and soybean peptides were effective in promoting the utilization of free fatty acids and improving VO2max exhaustive cycling test performance in humans. In a previous Systematic Review and Meta-analysis, Kressler et al. reported that quercetin supplementation could improve human endurance exercise capacity (VO2max and endurance exercise performance) (80). In our subgroup analysis, it is found that a greater increase in gait speed is expected in patients who do not regularly exercise compared with those who do. Similar to these findings, it was also reported that the dietary flavonoid quercetin could increase VO2max and endurance capacity, especially for people without taking exercise training (81). Clinically, this apparent increase in fitness without physical training may have implications beyond enhancing health promotion and illness prevention performance. VO2max is not only limited to oxygen delivery by the cardiovascular system but is also influenced by muscle mitochondrial oxidative capacity. Skeletal muscle, especially type I muscle fibers, is abundant in mitochondria generating the energy and oxygen continuously required for physical activity. Recent evidence has proven that lower mitochondrial capacity and efficiency are associated with reduced physical performance in age-related Sarcopenia (82). Flavonoids could enhance muscle mitochondrial function through diverse signaling pathways. Davis et al. found that quercetin could induce mitochondrial biogenesis in skeletal muscle by overexpression of the transcriptional coactivators sirtuin 1 (SIRT1) and peroxisome proliferator-activated receptor-γ coactivator (PGC1α) (83). Moreno et al. (84) also found that epicatechin exhibited greater increases in mitochondrial protein expression and indices of mitochondrial content through NO-dependent and Nrf2 pathways. In addition, various flavonoids have been reported to promote the switch of mitochondrial-rich type I fibers in skeletal muscle as discussed above (65, 66), thus improving aerobic capacity. Our subgroup analysis conducted by complications with Sarcopenia showed that flavonoids effectively improved the Timed-Up and Go for Sarcopenia population. The underlying mechanism is probably that the muscle fibers reduction and transformation in severe Sarcopenia is revered by flavonoid supplementation.

Concerning the clinical practice, most of the supplementations in this study are isoflavones or isoflavone-rich foods, and the mean isoflavones intake ranges from about 50–135 mg/d for 8–24 weeks. Fan et al. in their review of dose-response analysis showed that an increment of 0.5 mg/d isoflavones, 5 mg/d flavones, 25 mg/d flavanols, 50 mg/d anthocyanins, 100 mg/d proanthocyanins, were associated with a 5 % reduction in coronary heart disease risk, respectively (85). However, evidence for flavonoids against Sarcopenia risk is quite scarce. The non-standardized flavonoids intervention and the uncategorizable outcomes made specific investigations into optimal composition, dosage and duration impossible in the current review.

There are several strengths of this review. To the best of our knowledge, this is the first systematic review based on RCTs to determine the impact of flavonoid intake on muscle mass, muscle strength, and physical performance separately in middle-aged to older adults with or without Sarcopenia. Furthermore, we provided a valuable quantitative analysis of the current research and performed subgroup analyses and found differential benefits in non-exercise and sarcopenic populations. It put forward some clinical implications. Thirdly, we also applied GRADE to qualify our level of evidence. Using GRADE, we show that, although statistically significant, some of our findings have been downgraded in terms of certainty, noting that study design issues preclude further conclusions. The main reasons for downgrading the certainty of evidence were increased risk of bias, mainly in the blinding domains, and for some subgroup analyses, the low number of subjects in each respective group.

There are also notable limitations that should be recognized. Firstly, while a comprehensive search for four electronic databases has been undertaken, it is possible that some RCTs meeting the criteria of this review were overlooked, which may be present in gray literature, such as conference proceedings, or may have been published in another language. In addition, many of the RCTs discussed in this review utilized small sample sizes. Furthermore, the current meta-analysis failed to perform dose–response analysis hindering drawing a reliable conclusion on the optimal dose and duration. Meanwhile, we did not analyze the separate effects of different subclasses of flavonoids, which may introduce category errors. Therefore, the specific type, dosage, and duration of flavonoid supplementation to improve Sarcopenia-related parameters need to be further explored. Finally, although our quantitative test for publication bias was insignificant, careful consideration should be given when interpreting the results because some of the measured outcomes included relatively few studies with the potential risk of bias.

## Conclusions

Our results suggest that significant improvements were primarily observed in appendicular skeletal muscle mass and 6-min walk distance after flavonoid supplementation. Results of subgroup analyses showed that flavonoids had more significant effects on appendicular muscle mass and Timed-Up and Go test in the Sarcopenia group, with a better impact on skeletal muscle mass and gait speed in the non-exercise group. However, no significant differences were found between the effects of the flavonoids on muscle strength.

## Data availability statement

The original contributions presented in the study are included in the article/Supplementary material, further inquiries can be directed to the corresponding author.

## Author contributions

Conceptualization: YunL. Design: YuL. Supervision, critical review, and funding acquisition: YanL and RT. Materials, data collection and processing, analysis and interpretation, literature search, and writing manuscript: YuL and YunL. All authors have read and agreed to the published version of the manuscript.

## Funding

This research was funded by the Research Program of Sports Bureau of Guangdong Province (Grant Number GDSS2020M003), City-School Joint Program of Guangzhou Science and Technology Bureau (Grant Number 202201020033), and Research-oriented Hospital Program of Guangzhou (Grant Number 2022RHPG05). These funding sources had no role in the design, methods, data collection, analysis, or preparation of this manuscript.

## Conflict of interest

The authors declare that the research was conducted in the absence of any commercial or financial relationships that could be construed as a potential conflict of interest.

## Publisher's note

All claims expressed in this article are solely those of the authors and do not necessarily represent those of their affiliated organizations, or those of the publisher, the editors and the reviewers. Any product that may be evaluated in this article, or claim that may be made by its manufacturer, is not guaranteed or endorsed by the publisher.
