# Supplementary material for: Effect of flavonoids on skeletal muscle mass, strength and physical performance in middle-aged and older adults with or without Sarcopenia: A meta-analysis of randomized controlled trials
Source: Front Nutr. 2022 Oct 10;9:1013449. doi: 10.3389/fnut.2022.1013449 (PMC9589257; doi:10.3389/fnut.2022.1013449)
Supplement: Supplementary file 1 [file Table_1.DOCX]

Supplementary Material

# Supplementary Table 1. Searching query of four databases.

| **Database name** | **Query** |
| --- | --- |
| **PubMed** | #1: ((("Aged"[Mesh]) OR ("Middle Aged"[Mesh])) OR (Elderly)) OR (Middle Age)  #2: ((((("Sarcopenia"[Mesh]) OR (Sarcopenias)) OR (sketal muscle mass)) OR (appendicular muscle mass)) OR (muscular atrophy)) OR (lean body mass)  #3 (((((((((("Hand Strength"[Mesh]) OR (Grip Strength)) OR (Strength, Hand)) OR (Strength, Grip)) OR (Hand Grip Strength)) OR (Grip Strength, Hand)) OR (Strength, Hand Grip)) OR (Grip)) OR (Grips)) OR (Grasp)) OR (Grasps)  #4: ((((((((((((("Physical Functional Performance"[Mesh]) OR (Functional Performance, Physical)) OR (Functional Performances, Physical)) OR (Performance, Physical Functional)) OR (Performances, Physical Functional)) OR (Physical Functional Performances)) OR (Functional Performance)) OR (Functional Performances)) OR (Performance, Functional)) OR (Performances, Functional)) OR (Physical Performance)) OR (Performance, Physical)) OR (Performances, Physical)) OR (Physical Performances)  #5: (((((("Flavonoids"[Mesh]) OR (Anthocyanins)) OR (Flavan-3-ols)) OR (Flavanones)) OR (Flavones)) OR (Flavonols)) OR (Isoflavones)  #6: #2 OR #3 OR #4  #7: #1 AND #5 AND #6 |
| **Embase** | #1: 'aged'/exp OR 'aged' OR 'middle aged'/exp OR 'middle aged'  #2: 'flavonoid'/exp OR 'flavonoid' OR 'anthocyanin'/exp OR 'anthocyanin' OR 'flavanone derivative'/exp OR 'flavanone derivative' OR 'flavone derivative'/exp OR 'flavone derivative' OR 'flavonol derivative'/exp OR 'flavonol derivative' OR 'isoflavone derivative'/exp OR 'isoflavone derivative' OR 'flavan 3 ol'/exp OR 'flavan 3 ol'  #3: 'sarcopenia'/exp OR 'sarcopenia' OR 'muscle mass'/exp OR 'muscle mass' OR 'grip strength'/exp OR 'grip strength' OR 'hand strength'/exp OR 'hand strength' OR 'physical performance'/exp OR 'physical performance'  #4: #1 AND #2 AND #3 |
| **Cochrane Library** | #1: MeSH descriptor: [Aged] explode all trees  #2: MeSH descriptor: [Middle Aged] explode all trees  #3: ((Elderly) OR (Middle Age)):ti,ab,kw  #4: MeSH descriptor: [Flavonoids] explode all trees  #5: (Anthocyanins or Flavanones or Flavones or Flavonols or Isoflavones):ti,ab,kw  #6: MeSH descriptor: [Sarcopenia] explode all trees  #7: MeSH descriptor: [Muscular Atrophy] explode all trees  #8: MeSH descriptor: [Hand Strength] explode all trees  #9: MeSH descriptor: [Physical Functional Performance] explode all trees  #10: ((muscle mass) OR (appendicular muscle mass) OR (lean body mass)):ti,ab,kw  #11: (hand-grip strength OR dynapenia OR physical performance):ti,ab,kw  #12: #1 OR #2 OR #3  #13: #4 OR #5  #14: #6 OR #7 OR #8 OR #9 OR #10 OR #11  #15: #12 AND #13 AND #14 |
| **Web of Science** | #1: (TS=(aged OR Middle aged)) OR AB=(Elderly OR Middle Age)  #2: (TS=(Flavonoids)) OR AB=(Anthocyanins OR Flavan-3-ols OR Flavanones OR Flavones OR Flavonols OR Isoflavones)  #3: (TS=(Sarcopenia OR muscle mass OR Hand Strength OR physical performance)) OR AB=(Sarcopenias OR appendicular muscle mass OR muscular atrophy OR lean body mass OR hand-grip strength OR dynapenia)  #4: #1 AND #2 AND #3 |
